# Association of serum cystatin C level with coronary atherosclerotic plaque burden: a comprehensive analysis of observational studies and genetic study

**DOI:** 10.1186/s12872-023-03506-2

**Published:** 2023-10-10

**Authors:** Jun Chen, Jiayi Shen, Yuesong Pan, Jing Jing, Yongjun Wang, Tiemin Wei, Lingchun Lyu

**Affiliations:** 1 Department of Cardiology, Lishui Central Hospital, the Fifth Affiliated Hospital of Wenzhou Medical university, Lishui, 323000 Zhejiang China; 2 Zhejiang Chinese Medical University, Hangzhou, 310000 Zhejiang China; 3 Department of Neurology, Beijing Tiantan Hospital, Capital Medical University, Beijing, China; 4 China National Clinical Research Center for Neurological Diseases, Beijing, China

**Keywords:** Cystatin C, Coronary artery disease, Plaque burden, Population-based study, Genetics, Mendelian randomization

## Abstract

**Background and Aims:**

Epidemiological studies show that high circulating cystatin C is associated with risk of cardiovascular disease (CVD), independent of creatinine-based renal function measurements. However, the relationship between serum cystatin C level and coronary atherosclerotic plaque burden is limited. We aimed to evaluate the relationship between circulating cystatin C and coronary atherosclerotic plaque burden.

**Methods:**

This study was a cross-sectional study based on China community population. Measurements of plaque burden were based on the segment-involvement score (SIS) and segment stenosis score (SSS), which derived from the Coronary Artery Tree Model Depicting Coronary Artery Plaque Scores. Logistic regression model was used to demonstrate the association between cystatin C level and coronary artery plaque burden. Mendelian randomization (MR) analyses were conducted to assess the causal effect of cystatin C level on coronary atherosclerosis risk.

**Results:**

A total of 3,043 objects were included in the present study. The odds risks (OR) of severe plaque burden in the highest serum cystatin C levels (OR: 2.50; Cl:1.59–3.91; P < 0.001) and medium-level cystatin C levels (OR: 1.86; 95% Cl: 1.21–2.88; P = 0.005) were significantly higher after fulled adjusted confounders compared with the lowest levels of serum cystatin C by SSS. The MR analysis showed that genetic predicted cystatin C levels was associated with an increased risk of coronary atherosclerosis (OR, 1.004; 95% CI, 1.002–1.006, P < 0.001) .

**Conclusion:**

Elevated serum cystatin C levels were associated with coronary atherosclerotic plaque burden. Cystatin C levels had a causal effect on an increased risk of coronary atherosclerosis at the genetic level.

**What is already known on this topic?:**

Coronary artery disease is currently the most common cardiovascular disease and the leading global cause of mortality. Previous studies reported that higher serum cystatin C levels were associated with an increased risk for future cardiovascular events, independent of the normal creatinine levels or estimated glomerular filtration rate (eGFR) values. The presence of high-risk coronary atherosclerotic plaque burden is associated with increased risk of cardiovascular events. However, the association between serum cystatin C and coronary atherosclerotic plaque burden is not very clear.

**What this study adds?:**

Our study demonstrated that the elevated serum cystatin C levels were associated with coronary atherosclerotic plaque burden. In addition, we found that serum cystatin C levels had a causal effect on an increased risk of coronary atherosclerosis at the genetic level.

**How this study might affect research, practice or policy?:**

Current research finds that serum cystatin C levels were associated with coronary atherosclerosis. The metabolic pathway of cystatin C could be a target for new therapies against CAD.

**Supplementary Information:**

The online version contains supplementary material available at 10.1186/s12872-023-03506-2.

## Introduction

Due to the aging global population, coronary artery disease (CAD) is currently the most common cardiovascular disease worldwide [1]. It has been found to be the leading cause of death in both developed and developing countries [2]. Atherosclerotic plaque is the essential pathological feature of CAD and is closely associated with future cardiovascular events [3]. The extent of atherosclerotic plaque is often quantified as plaque burden, which is the percentage of plaque area within the entire vessel area [4]. Risk of adverse events from coronary artery disease (CAD) starts to rise with the presence of mild atherosclerotic disease and gradually increases with the extent of atherosclerotic plaque burden [5]. Atherosclerotic plaque burden could be directly visualized and quantified noninvasively by coronary computed tomography angiography [6]. Recently, Palanca A et al. found that plaque burden of subclinical atherosclerosis is the strongest predictor of future cardiovascular events in diabetic individuals with chronic kidney disease [7]. Accordingly, measurements of plaque burden and composition determined by coronary computed tomography angiography (CTA) have been proven to predict future cardiovascular events well [8–10].

Cystatin C is a important cysteine protease inhibitor which has essential function in maintaining vascular function and structure especially through regulate cathepsins S and cathepsins K [11]. Previous studies reported that higher serum cystatin C levels were associated with an increased risk for future cardiovascular events, independent of the normal creatinine levels or estimated glomerular filtration rate (eGFR) values [12–14], while, the exact mechanism remains unclear. Subclinical coronary atherosclerotic plaque burden is important pathological processes of CAD, whether the aggravation of coronary plaque burden is accompanied by the increase of serum cystatin C level is a topic worth exploring. However, there were no studies reported the association between serum cystatin C level and coronary atherosclerotic plaque burden. Given that it is difficult to evaluate the causality of serum cystatin C level on coronary atherosclerosis for the potential confounding interference that exist in observational studies. We used Mendelian Randomization (MR) analysis to evaluate causality of serum cystatin C level on coronary atherosclerosis by using genetic variants as instrumental variables for risk factors. Therefore, the purpose of this study is to investigate the association between serum cystatin C levels and coronary atherosclerotic plaque burden, and provide more references for the prevention of coronary atherosclerotic heart disease in the future.

## Methods

### Study design and clinical data

The study data were derived from the PRECISE study (NCT03178448) and the protocol has been reported in previous study [15]. We extracted the general characteristics (e.g., smoking, drinking, hypertension, diabetes, body mass index, waistline, hipline, blood pressure, etc.) and laboratory data (plasma levels of cystatin C, total cholesterol (TC), triglyceride (TG), low-density lipoprotein cholesterol (LDL-C), high-density lipoprotein cholesterol (HDL-C), creatinine, uric acid (UA), urea nitrogen, lipoprotein(a) (LP(a)), homocysteine, glycosylated hemoglobin) of participants. Hypertension is defined as either systolic blood pressure (SBP) ≥ 140 mmHg or diastolic blood pressure (DBP) ≥ 90 mmHg at least three times, self-reported hypertension previously diagnosed by a physician or on a prescription of antihypertensive chemotherapy. Diabetes is defined as a self-reported diabetes previously diagnosed by a physician or current use of anti-diabetic agents or fasting plasma glucose ≥ 7.0 mmol/L or 2-hour postload glucose ≥ 11.1 mmol/L or HbA1c ≥ 6.5% [13]. Body Mass Index (BMI) is calculated as weight (kg) divided by height^2^ (m^2^).

### Coronary computed tomography angiography examination

Participants with a potential risk of using contrast media were excluded, and the remained participants were requested to underwent CTA for major arteries of the body (coronary artery, subclavian artery, renal and iliofemoral arteries). All scans and readings were performed by clinicians blinded to the trial. Plaque burden was defined as the sum of calcified plaque burden and non-calcified plaque burden. Measurements of plaque burden were based on the segment-involvement score (SIS) and segment stenosis score (SSS), which derived from the Coronary Artery Tree Model Depicting Coronary Artery Plaque Scores [16]. The segment-involvement score divided the coronary arteries into 16 segments [16]. Given that some blood vessels were too small and had deviation in image recognition in actual research, we only included nine main segments for final research. The nine main segments of coronary artery include left main, left anterior descending, diagonal branch, circumflex, obtuse marginal, septal branch, right coronary artery, posterial descending branch, posterior branches of left ventricular. The segment stenosis scores were used as a measure of overall coronary artery plaque extent. Each coronary segment was graded as having no plaque to severe plaque (i.e., scores from 0 to 3) based on the inner diameter of coronary artery. Then the extent scores of all nine individual segments were summed to yield a total score ranging from 0 to 27. Previous studies reported that the significant coronary atherosclerotic burden which based on 16 segments of Coronary Artery Tree Model, was defined using prognostically validated cutoffs: SIS > 4 [17], or SSS > 5 [18]. Given that there were nine individual segments in our study, thus, we regarded SSS ≥ 4 were represented severe coronary artery plaque burden, SSS ≥ 1 but < 4 were represent non-severe disorder. Two raters reconstructed and analyzed the CTA data at a cardiac image-viewing workstation.

### Statistical analysis

Continuous variables that exhibited a normal distribution were documented as the mean ± standard deviation (SD). Otherwise, they were documented as medians with upper and lower quartiles. Categorical variables were documented as frequencies with percentages. Group comparisons were pooled using Chi-square, 1-way ANOVA, and Kruskal-WallisH tests. For each clinical outcome measures, three multivariate logistic regression models were constructed on the basis of cystatin C group inclusion according to tertiles. The first tertile was treated as the reference group. In the model I, covariates were adjusted for age, gender, BMI, diabetes, smoking, waistline and hipline. In the model II, we adjusted for creatinine, UA, homocysteine, cholesterol, LDL-C, HDL-C, triglyceride, HbA1c. In the model III, we further adjusted for covariates age, gender, BMI, diabetes, smoking, hypertension, creatinine, homocysteine, UA, LDL-C, HbA1c. Additionally, we performed a subgroup analysis for further investigating the association between serum cystatin C levels and coronary atherosclerotic plaque burden based on the model III. All tests were 2-tailed tests, and P ≤ 0.05 was considered statistically significant. Statistical analyses were performed using R versions 3.4.2 (R Foundation for Statistical Computing, Vienna, Austria).

### Data sources of mendelian randomization

Summary level data on the associations of cystatin C was obtained from recently published a large-scale genome-wide association studies (GWAS) [33]. We selected summary statistics for serum cystatin C. A total of 358 SNPs were genome-wide significant with cystatin C ( p < 5 × 10^− 8^) (Supplementary material). The summary statistics data for coronary atherosclerosis was derived from the GWAS database (https://gwas.mrcieu.ac.uk/datasets/ukb-d-I9_CORATHER/;ICD:“ukb-d-I9-CORATHER”). Studies contributing data to these GWAS meta-analyses had received ethical approval from relevant institutional review boards. In the present study, we only made use of the summarized data from these studies; hence, no additional ethics approval was required.

### Statistical analysis

The primary MR analysis was performed using the fixed effects inverse variance weighted (IVW) and random effects inverse variance weighted (IVW) methods. As the presence of horizontal pleiotropy in IVW estimates, MR-Egger and weighted median were also conducted to test the robustness of the results. In the sensitivity analysis, the heterogeneity and pleiotropy of individual SNPs were evaluated using IVW methods with Cochran’s Q statistics and MR Egger intercept, respectively. Also, a leave-one-out analysis was performed to evaluate the robustness of MR analysis results through any outlier SNP. All statistical analyses were undertaken using the “TwoSampleMR” package in R version 3.4.2 (R Foundation for Statistical Computing, Vienna, Austria) and a two-tailed p value < 0.05 was considered statistically significant.

## Results

### Characteristics of the study population

A total of 3,043 participants (1,413 males and 1,630 females) with an average age of 61.1 ± 7.5 years old were included in our study. The mean SIS and SSS was highest in tertiles 3 and lowest in tertiles 1 (P < 0.001). Atherosclerotic plaques appeared in 1186 (38.97%) individuals. Among the objects, 900 objects had mild-to-moderate plaque burden with the prevalence of 29.6%, and 286 objects had severe plaque burden with the prevalence of 9.4% according to the SSS. The prevalence of severe plaque burden according to SSS among each cystatin C group was 3.4% (33/979), 8.4% (85/1016), and 16% (168/1048), respectively. Mean age, BMI, waist and hip circumference, SBP, prevalence of men, hypertension, diabetes, dyslipidemia, stroke, smoking, creatinine, UA, BUN and Triglyceride significantly increased with serum cystatin C tertiles. By contrast, total cholesterol, and HDL cholesterol were significantly decreased with serum cystatin C tertiles. However, there were no significant difference in ankle brachial index between the various group. More information about the study population characteristics is shown in Table 1.

**Table 1:** Baseline Characteristics of Participants

variable	Q1 (n = 979) (0.52–0.85) mg/dL	Q2 (n = 1016) (0.86–0.99) mg/dL	Q3 (n = 1048) (1.00-7.35) mg/dL	P value
Age (years old)	58.2 ± 5.5	60.9 ± 6.4	64.3 ± 6.6	**< 0.001**
Man, n (%)	291(29.7)	469(46.2)	653(62.3)	**< 0.001**
BMI (kg/m^2^)	23.5 ± 3.0	23.7 ± 3.0	24.1 ± 3.1	**< 0.001**
SBP (mmHg)	127.5 ± 16.1	128.9 ± 16.3	131.2 ± 16.4	**< 0.001**
DBP (mmHg)	74.9 ± 8.9	75.1 ± 9.2	75.5 ± 9.0	0.3469
Hypertension (%)	325(33.2)	422(41.5)	557(53.1)	**< 0.001**
Diabetes (%)	186(19.0)	202(19.9)	270(25.8)	**0.0003**
dyslipidemia	378(38.6)	412(40.6)	479(45.7)	**0.0035**
Stroke	14(1.4)	23(2.3)	49(4.7)	**< 0.001**
renal_disorder	107(10.9)	121(11.9)	149(14.2)	0.0684
Smoking (%)	102(10.4)	212(20.9)	311(29.7)	**< 0.001**
Drinking (%)	171(17.5)	203(20.0)	194(18.5)	0.3500
waistline	84.8 ± 8.7	86.5 ± 8.6	88.7 ± 9.0	**< 0.001**
hipline	94.4 ± 6.3	95.0 ± 6.1	95.8 ± 6.4	**< 0.001**
Creatinine (umol/L)	57.9 ± 10.1	65.0 ± 11.2	76.4 ± 20.5	**< 0.001**
UA (umol/L)	303.0 ± 71.4	336.4 ± 75.0	378.0 ± 91.4	**< 0.001**
BUN (mmol/L)	5.5 ± 1.4	5.8 ± 1.4	6.2 ± 1.5	**< 0.001**
Cholesterol (mmol/L)	5.4 ± 1.0	5.3 ± 1.0	5.1 ± 1.0	**< 0.001**
LDL-C (mmol/L)	2.8 ± 0.8	2.8 ± 0.8	2.7 ± 0.8	**< 0.001**
HDL-C (mmol/L)	1.5 ± 0.3	1.4 ± 0.3	1.3 ± 0.3	**< 0.001**
LP(a) (mg/L)	60.0(32.0-146.0)	63.0(31.0-145.5)	64.0(33.0-159.0)	0.4178
Triglyceride (mmol/L)	1.7 ± 1.3	1.8 ± 1.2	1.9 ± 1.2	**0.0002**
Cys (mg/L)	0.8 ± 0.1	0.9 ± 0.0	1.2 ± 0.2	**< 0.001**
HbA1c	5.9 ± 0.9	5.9 ± 0.9	6.0 ± 1.0	**0.0001**
glu (mmol/L)	6.0 ± 1.6	5.9 ± 1.4	6.0 ± 1.7	0.6705
abil	1.1 ± 0.1	1.1 ± 0.1	1.1 ± 0.1	0.9851
abir	1.1 ± 0.1	1.1 ± 0.1	1.1 ± 0.4	0.9111
SIS (1–3)	299(30.5)	405(39.9)	503(48.0)	**< 0.001**
SIS (≥ 4)	15(1.5)	55(5.4)	88(8.4)	**< 0.001**
SSS (1–3)	238(24.3)	307(30.2)	355(33.9)	**< 0.001**
SSS (≥ 4)	33(3.4)	85(8.4)	168(16.0)	**< 0.001**

BMI: body mass index; SBP: systolic blood pressure; DBP: diastolic blood pressure; UA: uric acid; BUN: blood urea nitrogen; LDL: low density lipoprotein-Cholesterol; HDL: high density lipoprotein-Cholesterol; LP(a): lipoprotein(a); Cys: homocysteine; HbA1c: glycosylated hemoglobin; abil: ankle brachial index of left leg; abir: ankle brachial index of right leg; SIS: segment-involvement score; SSS: segment stenosis score

Quartiles of cystatin C, mg/dL

### The association of serum cystatin C levels with coronary atherosclerotic plaque burden

Serum cystatin C levels had a positive association with coronary atherosclerotic plaque burden. In the model I, after adjusted for the general information (age, gender, BMI, Diabetes, Smoking, waistline, hipline) of participants, we have not observed the significant association between serum cystatin C tertiles and mild-to-moderate coronary atherosclerotic plaque burden (cystatin C tertiles 3 vs. cystatin C tertiles 1: OR: 1.25, 0.99–1.57; P = 0.056), however, for the severe coronary atherosclerotic plaque burden (SSS ≥ 4), the risk of severe coronary atherosclerotic plaque burden (SSS ≥ 4) was significantly higher with increasing serum cystatin C tertiles than with the first serum cystatin C tertiles (cystatin C tertiles 2 vs. cystatin C tertiles 1: OR: 2.01, 1.30–3.08; P = 0.002; cystatin C tertiles 3 vs. cystatin C tertiles 1: OR: 2.95, 1.92–4.51; P < 0.001). In the model II, we only adjusted for the risk factors of laboratory examination (Creatinine, UA, Cholesterol, LDL-C, HDL-C, Triglyceride, homocysteine, HbA1c). Both mild-to-moderate and severe coronary atherosclerotic plaque burden were all significant associated with increasing serum cystatin C tertiles (P < 0.001). In the model III, after full adjustment for cardiovascular disease risk factors (age, gender, BMI, Diabetes, Hypertension, Creatinine, UA, LDL-C, HbA1c), the risk of severe coronary atherosclerotic plaque burden gradually increased in the second (OR: 1.86; 95% CI: 1.21–2.88; P < 0.001), and third serum cystatin C tertiles (OR: 2.50; 95% CI: 1.59–3.01; P < 0.001) compared with the first serum cystatin C tertiles (As shown in Table 2).

**Table 2:** logistic regression model analysis for research the association between Cystatin C and coronary atherosclerotic disorder

Variable	Crude model		Model I		Model II		Model III	
SSS(mild-to-moderate)	OR (95% CI)	P value	OR (95% CI)	P value	OR (95% CI)	P value	OR (95% CI)	P value
Q1 (0.52–0.85)	1.0 (ref)		1.0 (ref)		1.0 (ref)		1.0 (ref)	
Q2 (0.86–0.99)	1.46 (1.20–1.79)	**< 0.001**	1.19 (0.96–1.46)	0.114	1.40 (1.14–1.72)	**0.002**	1.20 (0.97–1.48)	0.103
Q3 (1.00-7.35)	2.01 (1.65–2.46)	**< 0.001**	1.25 (0.99–1.57)	0.056	1.80 (1.42–2.28)	**< 0.001**	1.31 (1.01–1.69)	**0.042**
SSS (severe)	OR (95% CI)	P value	OR (95% CI)	P value	OR (95% CI)	P value	OR (95% CI)	P value
Q1 (0.52–0.85)	1.0 (ref)		1.0 (ref)		1.0 (ref)		1.0 (ref)	
Q2 (0.86–0.99)	2.92 (1.93–4.43)	**< 0.001**	2.01 (1.30–3.08)	**0.002**	2.53 (1.65–3.86)	**< 0.001**	1.86 (1.21–2.88)	**0.005**
Q3 (1.00-7.35)	6.87 (4.65–10.14)	**< 0.001**	2.95 (1.92–4.51)	**< 0.001**	4.81 (3.14–7.35)	**< 0.001**	2.50 (1.59–3.91)	**< 0.001**

OR: Odds ratio; CI: confidence interval. Model was derived from logistic regression model. Crude model adjusted for: none

Model I adjusted for: age, gender, BMI, Diabetes, Smoking, waistline, hipline

Model II adjusted for: Creatinine, UA, Cholesterol, LDL-C, HDL-C, Triglyceride, HbA1c, homocysteine

Model III adjusted for: age, gender, BMI, Diabetes, Smoking, Hypertension, homocysteine, Creatinine, UA, LDL-C, HbA1c.

SIS: segment-involvement score; SSS: segment stenosis score

### Subgroup analyses

We observed cystatin C level was associated with severe plaque burden without significant interaction effect with sex, and diabetes. However, among the objects without hypertension, BMI < 24, renal disorder, and hyperuricemia, there were still lack of evidences showed that cystatin C level was significantly associated with severe plaque burden. Age has a special effect on cystatin C. It was observed that cystatin C was significantly associated with severe plaque burden in 55–65 years old objects, while only higher levels of cystatin C (Q3) was significantly associated with severe plaque burden in the objects who over 65 years old. Nevertheless, there was no significant association between serum cystatin C level and severe plaque burden in the objects who under 55 years old. The subgroup analyses results about serum cystatin C level and severe plaque burden as shown in Table 3.

**Table 3:** Subgroup analysis of the relationship between cystatin C and coronary atherosclerotic disorder

Characteristic	Group 1 (Ref)			Group 2		Group 3	
				OR(95%CI)	P value	OR(95%CI)	P value
Age (years old)							
< 55	Ref	SSS (sev)		2.99 (0.97–9.18)	0.056	2.52 (0.60-10.56)	0.205
55–65	Ref	SSS (sev)		2.35 (1.26–4.39)	**0.007**	4.35 (2.18–8.71)	**< 0.001**
≥ 65	Ref	SSS (sev)		1.62 (0.76–3.45)	0.209	2.48 (1.81–5.20)	**0.016**
Gender							
Male	Ref	SSS (sev)		2.11 (1.09–4.09)	**0.027**	2.06 (1.03–4.09)	**0.040**
Female	Ref	SSS (sev)		1.54 (0.84–2.80)	0.162	3.45 (1.86–6.37)	**< 0.001**
Diabetes							
Yes	Ref	SSS (sev)		3.61 (1.48–8.81)	**0.005**	4.88 (1.94–12.25)	**< 0.001**
NO	Ref	SSS (sev)		1.44 (0.87–2.40)	0.157	2.14 (1.24–3.69)	**0.007**
Hypertension							
Yes	Ref	SSS (sev)		1.76 (1.01–3.07)	**0.048**	2.85 (1.62-5.00)	**< 0.001**
NO	Ref	SSS (sev)		1.83 (0.90–3.74)	0.097	1.78 (0.81–3.93)	0.155
BMI, kg/m^2^							
< 24	Ref	SSS (sev)		1.66 (0.39–6.99)	0.490	1.82 (0.38–8.69)	0.456
≥ 24	Ref	SSS (sev)		1.88 (1.19–2.97)	**0.007**	2.57 (1.60–4.12)	**< 0.001**
Renal disorder							
Yes	Ref	SSS (sev)		2.06 (0.49–8.61)	0.320	2.78 (0.67–11.57)	0.159
NO	Ref	SSS (sev)		1.89 (1.19-3.00)	**0.007**	2.60 (1.59–4.27)	**< 0.001**
hyperuricemia							
Yes	Ref	SSS (sev)		3.41 (0.71–16.27)	0.124	3.71 (0.80-17.19)	0.094
NO	Ref	SSS (sev)		1.78 (1.12–2.81)	**0.014**	2.55 (1.59–4.11)	**< 0.001**

SIS: segment-involvement score; SSS: segment stenosis score

### Results of mendelian randomization study

**Fig. 1:**
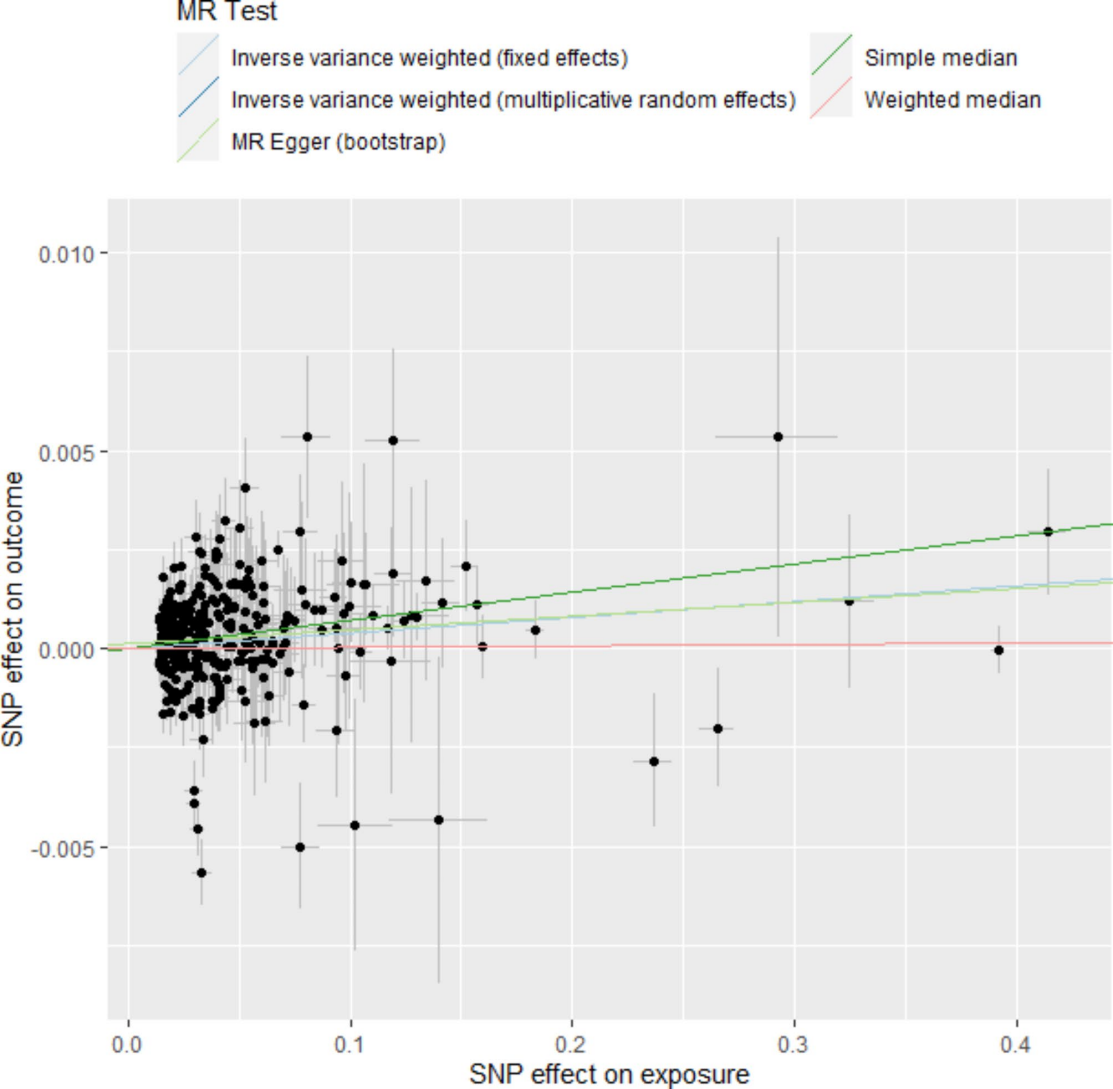
Scatter plot to visualize causal effect of serum cystatin C levels on coronary atherosclerosis. The slope of the straight line indicates the magnitude of the causal association

**Fig. 2:**
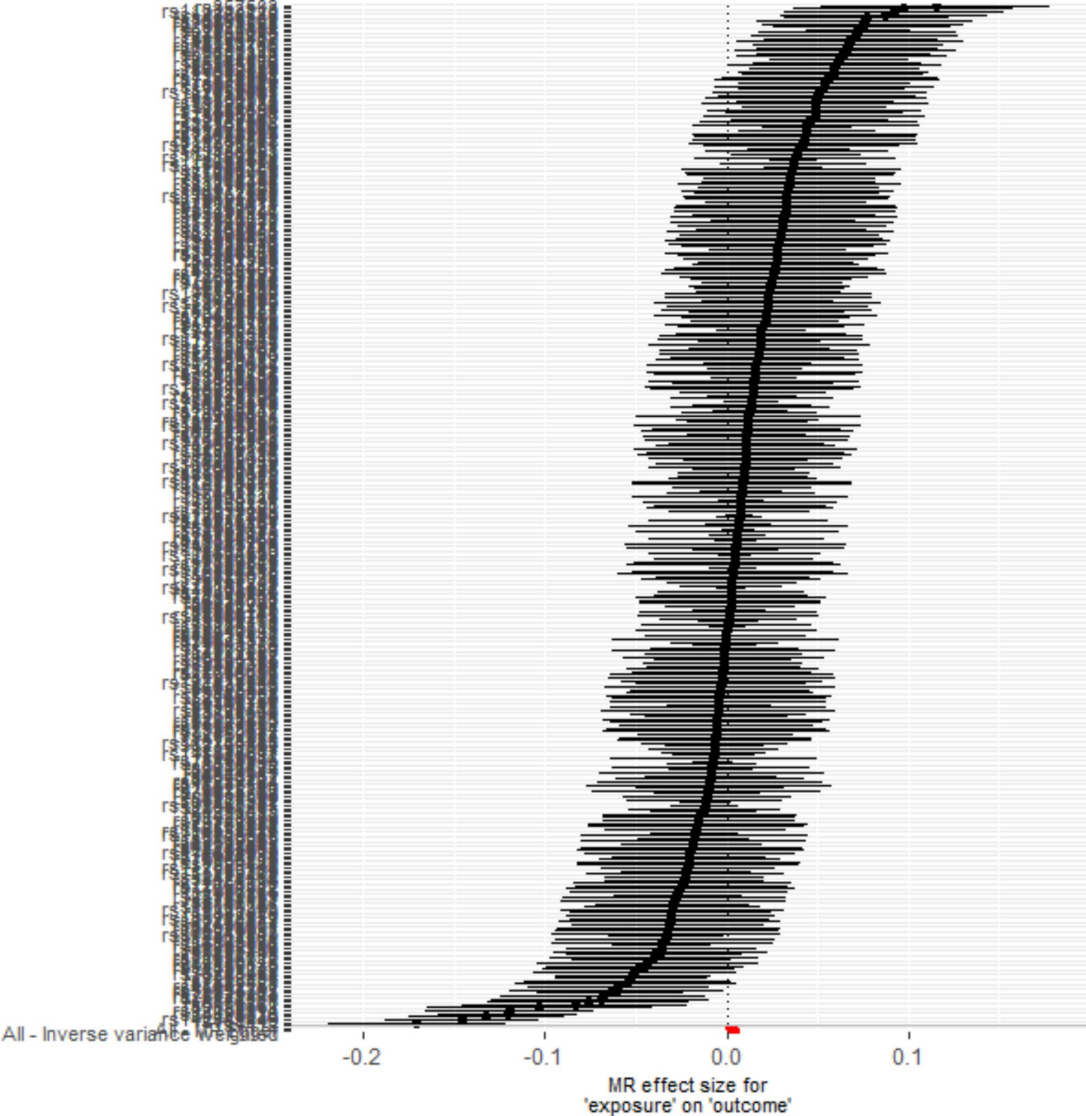
Fixed-effect IVW analysis of the causal association of serum cystatin C levels with coronary atherosclerosis. The black dots and bars indicated the causal estimate and 95% CI using each SNP. The red dot and bar indicated the overall estimate and 95% CI meta-analyzed by MR-Egger and fixed-effect inverse variance weighted method

**Fig. 3:**
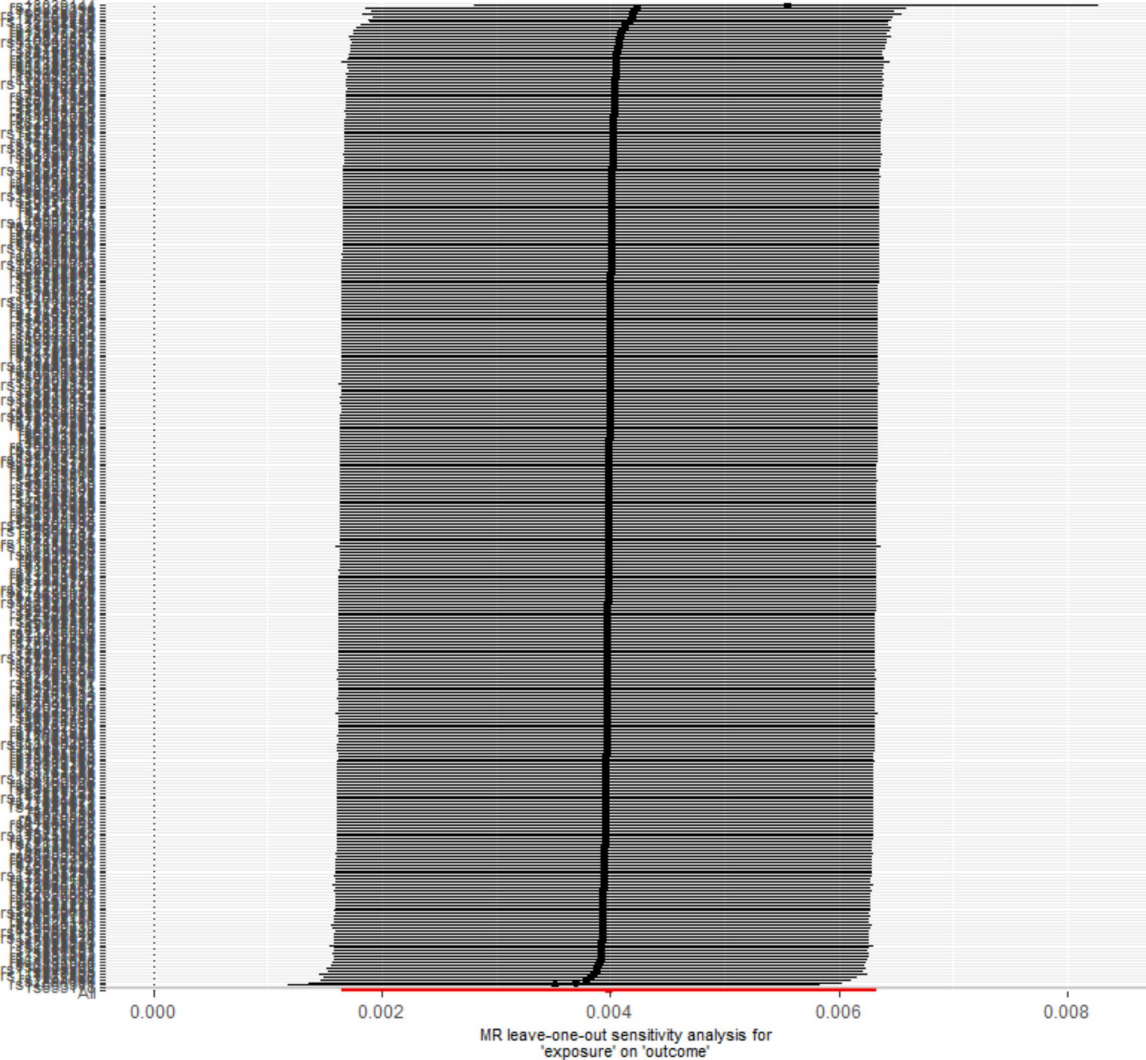
MR leave-one-out sensitivity analysis for serum cystatin C levels on coronary atherosclerosis. Circles indicate MR estimates for serum cystatin C levels on coronary atherosclerosis using inverse-variance weighted fixed-effect method if each SNP was omitted in turn

**Fig. 4:**
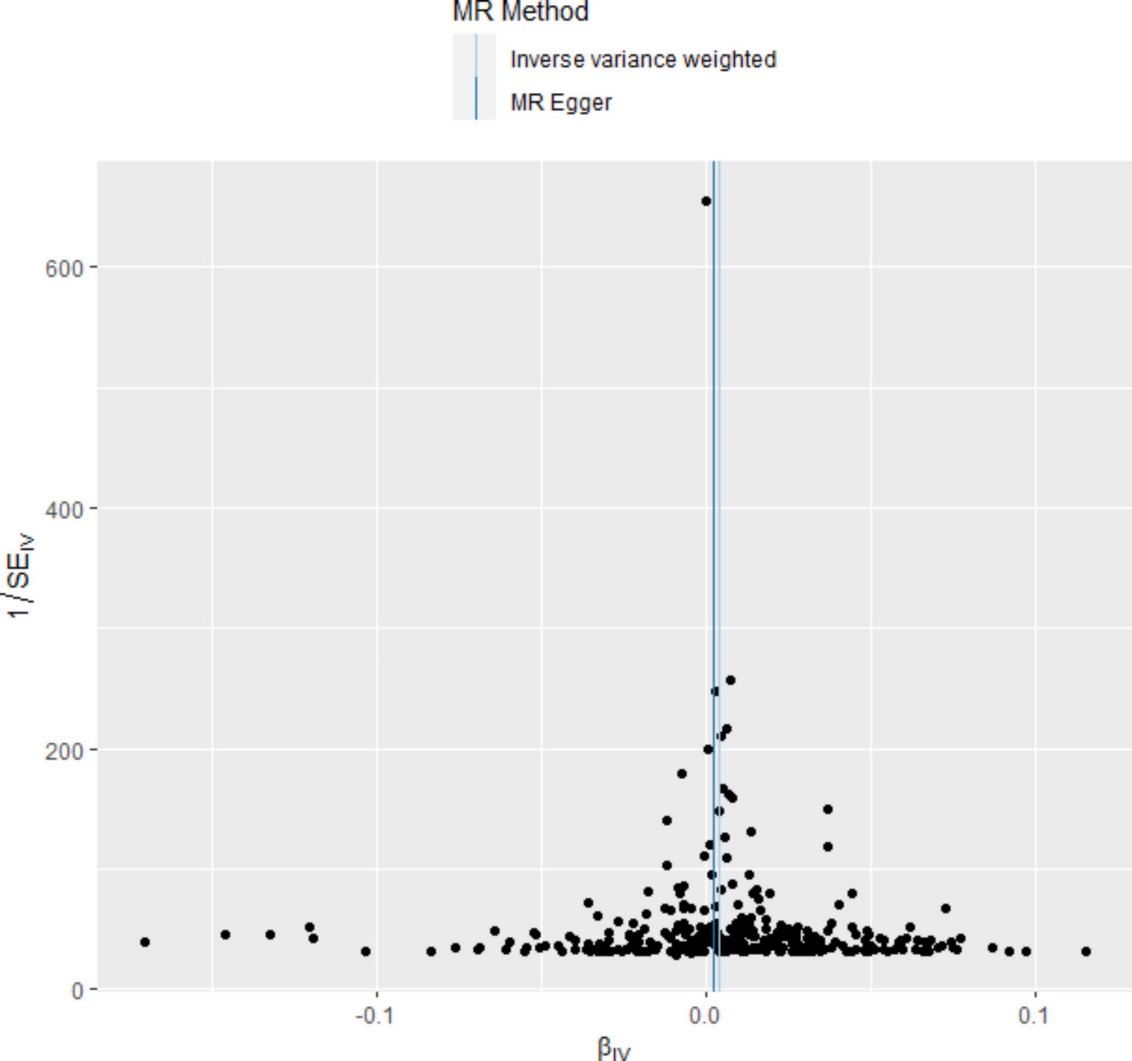
Funnel plot of genetic associations with serum cystatin C levels against causal estimates based on each genetic variant individually, where the causal effect is expressed in logs odds ratio of coronary atherosclerosis for each unit increase in serum cystatin C levels. The overall causal estimates (β coefficients) of serum cystatin C levels on coronary atherosclerosis estimated by inverse-variance weighted (light blue line) and MR-Egger (navy blue line) methods are shown

A total of 358 SNPs were genome-wide significant with serum cystatin C level. Ultimately, 358 SNPs as the instruments were included in the two-sample MR analysis after matching the coronary atherosclerosis data. Five MR analysis methods, inverse-variance weighted fixed-effect, inverse-variance weighted random-effect, simple median, weighted median, and MR-Egger (bootstrap) were performed to analyze the final results, as shown in Fig. 1. Both the fixed-effect and random-effect IVW models showed that serum cystatin C level was associated with an increased risk of coronary atherosclerosis (OR, 1.004; 95% CI, 1.002–1.006, P < 0.001; OR, 1.004; 95% CI, 1.002–1.006, P < 0.001), as shown in Fig. 2. Similar results was also observed using MR Egger (bootstrap) method (OR, 1.003; 95% CI, 1.001–1.006, P = 0.002), as shown in Table 4. The heterogeneity may exist in the IVW analysis (Q = 769.697, P < 0.001) and MR-Egger analysis (Q = 765.367, P < 0.001). MR-Egger regression showed no evidence of directional pleiotropic effect across the genetic variants (intercept, 0.0001; P = 0.166). The leave-one-out sensitivity analysis showed that the association between serum cystatin C level and coronary atherosclerosis was not substantially driven by any individual SNP (Fig. 3). Asymmetry in the funnel plot indicates directional horizontal pleiotropy, which can bias MR methods; however, the funnel plot and MR Egger regression test showed no evidence of asymmetry (Fig. 4).

**Table 4:** The association of serum cystatin C levels with coronary atherosclerosis risk using various methods

Method	Beta	SE	OR	95% CI	P value
IVW (random effects)	0.004	0.001	1.004	1.002–1.006	< 0.001
IVW (fixed effects)	0.004	0.001	1.004	1.002–1.006	< 0.001
Simple median	0.007	0.002	1.007	1.004–1.011	< 0.001
Weighted median	0.001	0.001	1.000	0.998–1.003	0.794
MR Egger (bootstrap)	0.003	0.001	1.003	1.001–1.006	0.002

IVW: inverse variance weighted

## Discussion

In this large-scale population-based study, we investigated the association between serum cystatin C level and coronary atherosclerotic plaque burden. To the best of our knowledge, it’s the first study to research the association between serum cystatin C level and coronary atherosclerotic plaque burden. Our results demonstrated that serum cystatin C was strongly associated with severe plaque burden independent of traditional cardiovascular disease predictors. Furthermore, cystatin C provides incremental information for the risk stratification of female objects age range 55 to 65 years old, accompany with hypertension or with higher BMI. In two-sample MR analyses, we found a potential association between genetic determinants of cystatin C level and coronary atherosclerosis.

Cystatin C is an endogenous cysteine proteinase inhibitor which is filtered by the renal glomerulus, and metabolized by the proximal tubule [19]. Compared with creatinine, it is less affected by age, sex, and lean muscle mass which regarded as a sensitive biomarker of renal function [20]. Clinical research has reported that higher levels of cystatin C may indicate the presence of any vulnerable plaque in CAD [21]. Cystatin C may associated with the destabilization and rupture of coronary artery plaque in the pathological processes of atherosclerosis, which were account for the high risk of cardiovascular events in highest cystatin C quartile [22]. The formation of coronary atherosclerotic plaque is closely related to inflammation [23]. Therefore, several studies proposed that cystatin C is related to inflammatory reaction, inflammatory status may contribute to changes in serum cystatin C levels [22–24]. Gao D et al. study revealed the level of cystatin C in active stage of systemic lupus erythematosus was higher than that in stable stage (P < 0.05), the increase degrees are negatively correlated with the inner diameter of brachial artery, which implied a correlation of serum cystatin C and vascular endothelial cell injury [25].

Previous studies included objects with acute coronary syndrome, objects with acute coronary syndromes tend to have a stronger inflammatory response than the general CAD population. Our study objects were community population with not serious inflammation, while, cystatin C was also strongly associated with severe coronary atherosclerotic plaque burden. Of note, Yamashita H et al. observed a significant association between cystatin C and cardio-ankle vascular index, which is a marker of early-stage arteriosclerosis [26]. Thereby, we speculated that cystatin C may be a product of medial destruction of coronary arteries. Previous study reported that the elastase-specific activity of the uninjured arterial extract was approximately half that of the atherosclerotic tissue extract, mainly due to cysteine proteases [27].

Cathepsin S–deficient mice with attenuated atherosclerosis provided convincing evidence for cysteine protease involvement in atherogenesis [28]. Subsequent researches reported that atherosclerosis relevant inflammatory cells and cytokines could stimulate the production of lysosomal cathepsins, and lead to the increased the plasma cystatin C concentrations [6–29]. These results revealed that there was a certain dynamic equilibrium in human tissues between cysteine proteases and cystatin C. Cysteine proteases increase with the aggravation of the activity of atherosclerosis, which contribute to an increase in cystatin C. Moreover, Ganda A et al. found that serum cystatin C levels were positively corrected with blood monocyte counts after adjusted traditional risk factors [30]. It suggests that the high blood monocyte counts may involve in the potential mechanisms that contribute to the strong relationship between cystatin C and cardiovascular risk [30]. In recent years, the relationship between cystatin C and cardiovascular diseases has been increasingly confirmed. Higher levels of cystatin C were associated with increased left ventricle mass and a concentric left ventricle hypertrophy phenotype independent of standard measurements of renal function [31]. Cystatin C protein was detected elevated in the plasma in cardiac injury by chronic administration of doxorubicin or in myocardial ischaemia by left anterior descending coronary artery occlusion, further analysis revealed an increase in cystatin C correlates with the inhibition of cathepsin B activity and accumulation of fibronectin and collagen I/III in myocardial tissue from the ischaemic area [32]. These studies provided compelling evidence that cystatin C plays an important role in the injury process of cardiovascular endothelial cells and cardiomyocytes.

In our study, it was found that cystatin C was closely correlated with multi-vessel lesions and coronary atherosclerotic plaque burden. Furthermore, serum cystatin C levels had a causal effect on an increased risk of coronary atherosclerosis at the genetic level. These results provide objective evidence that cystatin C may be involved in the early stage of CAD. However, more pathological mechanisms are still needs to be explored by related basic pathology studies. Further, our study subjects derive from cluster sampling of Lishui living communities (not occupational communities), they have similar demographics and medical histories as nationwide sample survey data. The representativeness of our study population allows us to better assess the prevalence of clinical or subclinical CAD.

Several study limitations should also be stated here. First, our sample size was not large enough from an epidemiological perspective, but this is the largest study ever conducted on the coronary atherosclerotic plaque burden. Second, most of the population being composed in our study were Han people, therefore, selection bias was inevitable. Given the many elements involved in CAD, we are cautious of the extension of our findings to different regions or races. Third, our study was a cross-sectional study based on community population. We demonstrated an association between cystatin C and coronary atherosclerotic plaque burden, but unable to reveal the causal relationship between them, this should be further research in the future.

### Conclusion

Elevated serum cystatin C levels were associated with coronary atherosclerotic plaque burden independent of traditional cardiovascular disease predictors as assessed by computed tomography coronary angiography. Furthermore, serum cystatin C levels had a causal effect on an increased risk of coronary atherosclerosis at the genetic level. The metabolic pathway of cystatin C could be a target for new therapies against CAD.

### Electronic supplementary material

Below is the link to the electronic supplementary material.

Supplementary Material 1

## Data Availability

Since this is an ongoing prospective cohort study, the data are not available in publicly database at present. However, data are available to researchers on request for purposes of reproducing the results or replicating the procedure by directly contacting the corresponding author.

## Declarations

### Statement of our study

We confirmed that all protocols were carried out in accordance with relevant guidelines and regulations; We confirmed that the informed consent was obtained from the all participants for the study.

## Abbreviations

coronary artery disease: CAD
computed tomography angiography: CTA
HbA1c: glycosylated hemoglobin
UA: Uric acid
OR: odds ratio
Cl: confidence interval
systolic blood pressure: SBP
diastolic blood pressure: DBP
BUN: blood urea nitrogen
total cholesterol: TC
triglyceride: TG
low-density lipoprotein cholesterol: LDL-C
high-density lipoprotein cholesterol: HDL-C
Cys: homocysteine
AUC: area under the curve
Lp(a): Lipoprotein (a)
OxPL: oxidized phospholipids
BMI: body mass index
segment-involvement score: SIS
segment stenosis score: SSS

## References

[1] Montalescot G, Sechtem U, Achenbach S, Andreotti F, Arden C, Budaj A et al. ESC guidelines on the management of stable coronary artery disease: the Task Force on the management of stable coronary artery disease of the European Society of Cardiology. Eur Heart J 2013;34(38)2949–3003 2013. 10.1093/eurheartj/eht296 23996286

[2] Malakar AK, Choudhury D, Halder B, Paul P, Uddin A, Chakraborty S (2019). A review on coronary artery disease, its risk factors, and therapeutics. J Cell Physiol.

[3] Libby P, Theroux P (2005). Pathophysiology of coronary artery disease. Circulation.

[4] Gerbaud E, Weisz G, Tanaka A, Luu R, Osman H, Baldwin G, Coste P, Cognet L, Waxman S, Zheng H (2020). Plaque burden can be assessed using intravascular optical coherence tomography and a dedicated automated processing algorithm: a comparison study with intravascular ultrasound. Eur Heart J Cardiovasc Imaging.

[5] Arbab-Zadeh A, Fuster V (2016). The risk continuum of atherosclerosis and its implications for defining CHD by coronary angiography. J Am Coll Cardiol.

[6] Knight EL, Verhave JC, Spiegelman D (2004). Factors influencing serum cystatin C levels other than renal function and the impact on renal function measurement. Kidney Int.

[7] Palanca A, Castelblanco E, Betriu A, Perpinan H, Soldevila B, Valdivielso JM, Bermudez-Lopez M, Puig-Jove C, Puig-Domingo M, Groop PH (2019). Subclinical atherosclerosis burden predicts cardiovascular events in individuals with diabetes and chronic kidney disease. Cardiovasc Diabetol.

[8] Motoyama S, Sarai M, Harigaya H (2009). Computed tomographic angiography characteristics of atherosclerotic plaques subsequently resulting in acute coronary syndrome. J Am Coll Cardiol.

[9] Motoyama S, Ito H, Sarai M (2015). Plaque characterization by coronary computed tomography angiography and the likelihood of acute coronary events in mid-term follow-up. J Am Coll Cardiol.

[10] Park H-B, Heo R, ó Hartaigh B (2015). Atherosclerotic plaque characteristics by CT angiography identify coronary lesions that cause ischemia: a direct comparison to fractional flow reserve. J Am Col Cardiol Img.

[11] van der Laan SW, Fall T, Soumare A, Teumer A, Sedaghat S, Baumert J, Zabaneh D, van Setten J, Isgum I, Galesloot TE (2016). Cystatin C and Cardiovascular Disease: a mendelian randomization study. J Am Coll Cardiol.

[12] Hoke M, Amighi J, Mlekusch W, Schlager O, Exner M, Sabeti S, Dick P, Koppensteiner R, Minar E, Rumpold H (2010). Cystatin C and the risk for cardiovascular events in patients with asymptomatic carotid atherosclerosis. Stroke.

[13] Luo J, Wang LP, Hu HF, Zhang L, Li YL, Ai LM, Mu HY, Kun-Wang (2015). Cystatin C and cardiovascular or all-cause mortality risk in the general population: a meta-analysis. Clin Chim Acta.

[14] Garcia-Carretero R, Vigil-Medina L, Barquero-Perez O, Goya-Esteban R, Mora-Jimenez I, Soguero-Ruiz C, Ramos-Lopez J (2017). Cystatin C as a predictor of cardiovascular outcomes in a hypertensive population. J Hum Hypertens.

[15] Pan Y, Jing J, Cai X (2020). PolyvasculaR evaluation for cognitive impairment and vaScular events (PRECISE)-a population-based prospective cohort study: rationale, design and baseline participant characteristics. Stroke Vasc Neurol.

[16] Min JK, Shaw LJ, Devereux RB, Okin PM, Weinsaft JW, Russo DJ, Lippolis NJ, Berman DS, Callister TQ (2007). Prognostic value of multidetector coronary computed tomographic angiography for prediction of all-cause mortality. J Am Coll Cardiol.

[17] Bittencourt SM, Hulten PE (2014). Prognostic value of nonobstructive and obstructive coronary artery disease detected by coronary computed tomography angiography to identify cardiovascular events. Circ: Cardiovasc Imag.

[18] Min JK, Shaw LJ (2007). Prognostic value of multidetector coronary computed tomographic angiography for prediction of all-cause mortality. JACC.

[19] Newman DJ, Cystatin C (2002). Ann Clin Biochem.

[20] Knight EL, Verhave JC, Spiegelman D, Hillege HL, de Zeeuw D, Curhan GC, de Jong PE (2004). Factors influencing serum cystatin C levels other than renal function and the impact on renal function measurement. Kidney Int.

[21] Doganer YC, Aydogan U, Aydogdu A, Aparci M, Akbulut H, Nerkiz P, Turker T, Cayci T, Barcin C, Saglam K (2013). Relationship of cystatin C with coronary artery disease and its severity. Coron Artery Dis.

[22] Patel D, Ahmad S, Silverman A, Lindsay J (2013). Effect of cystatin C levels on angiographic atherosclerosis progression and events among postmenopausal women with angiographically decompensated coronary artery disease (from the Women’s angiographic vitamin and estrogen [WAVE] study). Am J Cardiol.

[23] Hansson GK (2005). Inflammation, atherosclerosis, and coronary artery disease. N Engl J Med.

[24] Shlipak MG, Katz R, Cushman M, Sarnak MJ, Stehman-Breen C, Psaty BM, Siscovick D, Tracy RP, Newman A, Fried L (2005). Cystatin-C and inflammatory markers in the ambulatory elderly. Am J Med.

[25] Gao D, Shao J, Jin W, Xia X, Qu Y (2018). Correlations of serum cystatin C and hs-CRP with vascular endothelial cell injury in patients with systemic lupus erythematosus. Panminerva Med.

[26] Yamashita H, Nishino T, Obata Y, Nakazato M, Inoue K, Furusu A, Takamura N, Maeda T, Ozono Y, Kohno S (2013). Association between cystatin C and arteriosclerosis in the absence of chronic kidney disease. J Atheroscler Thromb.

[27] Sukhova GK, Shi GP, Simon DI, Chapman HA, Libby P (1998). Expression of the elastolytic cathepsins S and K in human atheroma and regulation of their production in smooth muscle cells. J Clin Invest.

[28] Sukhova GK, Zhang Y, Pan JH, Wada Y, Yamamoto T, Naito M, Kodama T, Tsimikas S, Witztum JL, Lu ML, Sakara Y, Chin MT, Libby P, Shi GP (2003). Deficiency of cathepsin S reduces atherosclerosis in LDL receptor-deficient mice. J Clin Invest.

[29] Evangelopoulos AA, Vallianou NG, Bountziouka V (2012). Association between serum cystatin C, monocytes and other inflammatory markers. Intern Med J.

[30] Ganda A, Magnusson M, Yvan-Charvet L, Hedblad B, Engström G, Ai D, Wang TJ, Gerszten RE, Melander O, Tall AR (2013). Mild renal dysfunction and metabolites tied to low HDL cholesterol are associated with monocytosis and atherosclerosis. Circulation.

[31] Patel PC, Ayers CR, Murphy SA, Peshock R, Khera A, de Lemos JA, Balko JA, Gupta S, Mammen PP, Drazner MH (2009). Association of cystatin C with left ventricular structure and function: the Dallas Heart Study. Circ Heart Fail.

[32] Xie L, Terrand J, Xu B, Tsaprailis G, Boyer J, Chen QM (2010). Cystatin C increases in cardiac injury: a role in extracellular matrix protein modulation. Cardiovasc Res.

[33] Sinnott-Armstrong N, Tanigawa Y, Amar D, Mars N, Benner C, Aguirre M, Venkataraman GR, Wainberg M, Ollila HM, Kiiskinen T, Havulinna AS, Pirruccello JP, Qian J, Shcherbina A, FinnGen, Rodriguez F, Assimes TL, Agarwala V, Tibshirani R, Hastie T, Ripatti S, Pritchard JK, Daly MJ, Rivas MA. Author Correction: Genetics of 35 blood and urine biomarkers in the UK Biobank. Nat Genet. 2021;53(11):1622. 10.1038/s41588-021-00956-2. Erratum for: Nat Genet. 2021;53(2):185–194. PMID: 34608296. 10.1038/s41588-020-00757-z PMC7867639 33462484

